# Communication, perception and behaviour during a natural disaster involving a 'Do Not Drink' and a subsequent 'Boil Water' notice: a postal questionnaire study

**DOI:** 10.1186/1471-2458-10-641

**Published:** 2010-10-25

**Authors:** Gabriella Rundblad, Olivia Knapton, Paul R Hunter

**Affiliations:** 1 Department of Education and Professional Studies, King's College London, Waterloo Bridge Wing, Waterloo Road, London SE1 9NH, UK; 2 School of Medicine, Health Policy and Practice, University of East Anglia, Norwich NR4 7TJ, UK

## Abstract

**Background:**

During times of public health emergencies, effective communication between the emergency response agencies and the affected public is important to ensure that people protect themselves from injury or disease. In order to investigate compliance with public health advice during natural disasters, we examined consumer behaviour during two water notices that were issued as a result of serious flooding. During the summer of 2007, 140,000 homes in Gloucestershire, United Kingdom, that are supplied water from Mythe treatment works, lost their drinking water for up to 17 days. Consumers were issued a 'Do Not Drink' notice when the water was restored, which was subsequently replaced with a 'Boil Water' notice. The rare occurrence of two water notices provided a unique opportunity to compare compliance with public health advice. Information source use and other factors that may affect consumer perception and behaviour were also explored.

**Method:**

A postal questionnaire was sent to 1,000 randomly selected households. Chi-square, ANOVA, MANOVA and generalised estimating equation (with and without prior factor analysis) were used for quantitative analysis.

**Results:**

In terms of information sources, we found high use of and clear preference for the local radio throughout the incident, but family/friends/neighbours also proved crucial at the onset. Local newspapers and the water company were associated with clarity of advice and feeling informed, respectively. Older consumers and those in paid employment were particularly unlikely to read the official information leaflets. We also found a high degree of confusion regarding which notice was in place at which time, with correct recall varying between 23.2%-26.7%, and a great number of consumers believed two notices were in place simultaneously. In terms of behaviour, overall non-compliance levels were significantly higher for the 'Do Not Drink' notice (62.9%) compared to the 'Boil Water' notice (48.3%); consumers in paid employment were not likely to comply with advice. Non-compliance with the general advice to boil bowser water was noticeably lower (27.3%).

**Conclusion:**

Higher non-compliance during the 'Do Not Drink' notice was traced to the public's limited knowledge of water notices and their folk beliefs about the protection offered from boiling water. We suggest that future information dissemination plans reduce reliance on official leaflets and maximise the potential of local media and personal networks. Current public health education programmes are recommended to attend to insufficient and incorrect public knowledge about precautionary actions.

## Background

During times of major public health emergencies, good communication between the emergency response agencies and the public in affected areas is vital [1]. Effective communication is particularly important when it is essential that people take steps to protect themselves from injury or disease. When looking at why people did not evacuate the city prior to the arrival of Hurricane Katrina, Brodie and colleagues [2] found that about one-third did not get the message and a further one-third heard the message but did not understand how to evacuate. They also reported that people who did not evacuate were predominantly from the poorest and most marginalised sections of society. Further, following terrorist attacks, good communication with the public is essential not only to reduce acute morbidity and mortality but also in mediating the social and psychological impact of terrorist attacks [3]. Indeed, it has been argued that poor communication may itself pose a risk to health by heightening anxiety and the development of somatoform disorders [4].

The experience of Hurricane Katrina reported by Brodie and colleagues [2] illustrates the main issues associated with risk communication during natural disasters. Firstly, getting the message to all the people including the poor and marginalised sections of society, and secondly, ensuring that the message is understood and leads to appropriate responses. Typically, risk communication is considered to contain four components: message, source, transmitter and receiver [e.g. [5]]. Original theories of risk communication drew upon the concept of the individual as a 'rational actor' who receives information from a knowledgeable authority source and then uses this information to manage and minimize their risk exposure [6]. Arguably, non-compliance is not necessarily driven by a lack of knowledge or an 'information gap' between official sources and the public. Rather it is a combination of individual and other societal factors that leads an individual to make active choices concerning their behaviour. These factors range from demographics, knowledge and previous experience of similar situations, and general health beliefs and attitudes towards risks and preventative actions, to the particular transmitter(s) used to convey the message to the public. Previous transmitter studies have highlighted the impact of media [7-9] and interpersonal contacts with health professionals [10]. Compliance studies have, however, found that whether consumers received the advice from a leaflet or some type of mass media did not impact compliance [11,12]. In September 2005, a category 3 hurricane, Hurricane Rita, made landfall on the Texas/Louisiana border, US. The chaos brought about by Hurricane Rita was such that word of mouth proved more useful than media channels [13].

On the 19^th^-20^th ^July 2007, the equivalent of two months of rain (125 mm) fell over Gloucestershire (total population 528,370) and neighbouring areas in the United Kingdom (UK), causing Britain's 'largest peacetime emergency since World War II' [[14]:vii]. There was widespread flash flooding and fluvial flooding of the River Severn and the River Avon. Approximately two days later, Mythe water treatment works (which is one of the main water works in Gloucestershire and is managed by Severn Trent Water (STW)) was flooded and Castle Mead electricity substation had to be shut down, leaving 140,000 homes (ca. 340,000-350,000 consumers) without mains water [15] and 48,000 homes without electricity [16]. While electricity was returned within 48 hours, consumers were left without drinking water for up to 17 days. No UK water company has ever experienced loss of supply on such a large scale before [15]. Before mains water was restored to normal, alternative supplies were provided. A total of 40 million bottles of water were distributed and an average of 3 million litres of water per day provided by mobile water tanks (bowsers). The provision of bowser and bottled water generally overlapped. Water restoration began on 27^th ^July, after confirmed delivery to all affected consumers of a non-standard 'Do Not Drink' notice devised by the regional health authority (Gloucestershire Primary Care Trust). The 'Do Not Drink' notice was replaced by a water industry standard precautionary 'Boil Water' notice seven days later, at which point the bowsers were withdrawn. On 7^th ^August, the water company (STW) declared the tap water safe to drink. The day before, the emergency phase of the Gloucestershire floods had been formally declared over and recovery begun [17].

When there is a risk that public health is threatened through contaminated drinking water, one of three standard notices may be issued: 'Boil Water' (= water safe to ingest after it has been boiled), 'Do Not Drink' (= water should not be ingested) and 'Do Not Use' (= water should not be used). While 'Boil Water' notices are relatively common, 'Do Not Drink' notices are rarely issued, and the combination of two notices is exceptionally rare [18,19]. 'Do Not Drink' notices are generally reserved to incidents where short-term exposure to contaminants is likely to have adverse health effects [20]; for the Mythe incident, the risk turned out to be minimal [15]. The issuing of a 'Do Not Drink' and a subsequent 'Boil Water' notice during the same natural disaster afforded us a unique opportunity to investigate compliance with public health advice and to investigate factors associated with risky and cautious behaviour to this advice (including the general advice always to boil bowser water before consumption).

## Method

### Study design and sample selection

A postal questionnaire study of 1,000 households supplied with mains water from Mythe waterworks and affected by loss of water and two subsequent water notices during summer 2007 was carried out in January-February 2009 (18 months after the incident). Assuming a response rate of 40%, 1000 questionnaires would yield a sample size of 400 which would give a standard error of less than 2.5%. Postcodes of affected households were provided by the Drinking Water Inspectorate. Full addresses were obtained using the Royal Mail Postcode Address File and 1,000 addresses were selected using a random number generator. Addresses were scrutinised and any businesses and schools were replaced with a further randomised selection of residential addresses.

Ethical approval was received from the King's College London Social Sciences, Humanities and Law Research Ethics Sub Committee.

### Questionnaire design

A questionnaire containing nine sections was constructed. A short section on demographics was followed by four sections that followed the chronological order of the incident: the initial period without mains water ('Water Loss'), the 'Do Not Drink' notice, the 'Boil Water' notice, and the time immediately after the water had been declared safe to drink ('Water Safe'). The sections for the four 'incident stages' contained questions about the respondents' uses of water straight from the tap (if any), uses of boiled water (if any), types of cold water used for drinking, the advice that they remembered receiving and the information sources from which they got their advice. Following these 'incident stages' sections, the questionnaire ended with several shorter sections concerning the use of temporary water supplies (in particular bowser water), use of information sources, information source preferences and previous experience and knowledge of water incidents. Mainly closed questions were employed. Open-ended questions were predominantly used towards the end of the questionnaire.

The questionnaire was piloted twice on undergraduate students from King's College London (N = 50), and minor revisions were made in order to improve clarity. The final questionnaire was sent out together with a detailed project description and a stamped, addressed return envelope. Non-responders were sent a reminder four weeks later.

### Coding

Where respondents ticked rather than ranked options on the four ranking questions, one tick alone was coded as rank one. If a respondent ranked several options and then ticked one further option, the ticked option was coded as their lowest rank. If only ticks were used, these were given the same rank (e.g., three ticks were ranked as 2). Between 46 and 63 of the participants used ticks. For 'tick only one' questions, multiple ticks were typically entered as inconclusive. However, for questions such as those asking what water notice participants remembered being in place during one of the incident stages, we coded multiple ticks as 'believed more than one advice was in place' in order to allow analysis of uncertainty. For questions where respondents selected 'other' and then qualitatively specified their answer, these were re-coded into the original categories where possible. Similarly for information sources, website usage was re-coded so that only sources that occur solely on the internet are in the category 'websites', while e.g., 'local radio' included listening, phoning and visiting the website of this source. Replies from between two and ten participants under the categories 'other' and/or 'websites' were re-coded into original categories. Open-ended questions were also quantified where possible; for example, occupations were translated into the binary categories 'yes, currently employed' and 'no, not currently employed'. Across all questions, non-responses were coded as missing data and inconclusive replies were generally excluded from further analysis.

### Analyses and hypotheses

The data were entered into Microsoft Access 2007 and then validated against the original questionnaires. For statistical comparison, data were transferred into SPSS version 16 and validated a second time, with the exception of demographic comparison which was performed using StatsDirect. Some questions were not (fully) answered by all respondents, thus sample size often varied between questions.

We hypothesised apriori that non-compliance with water advice would be higher for the 'Do Not Drink' phase compared to the 'Boil Water' one. In addition, we predicted that demographic factors (such as age, gender, home ownership and employment), prior beliefs/experiences, use of information sources, and practical issues (e.g. loss of electricity) could have had an effect on participants' perceptions and behaviours; however, as no formal hypotheses were defined apriori, statistical outcomes should be interpreted solely as indicators of the potential strength of association. Quantitative analysis is mainly descriptive. Inferential analysis was carried out using chi-square, ANOVA, and for repeated measures generalised estimating equations (with and without prior factor analysis). For analyses with multiple outcome variables, initial analysis was done using multivariate ANOVA with all possible outcome and predictor variables. For all analyses with multiple predictor variables, only those variables that were significant at the p < 0.2 level in single predictor models were included in the multiple predictor models. The least significant variable was then removed from the model until all predictor variables were significant at the p < 0.2 level. The value of the model in predicting each dependent variable was then derived from the tests of between subjects effects in the corrected model. Throughout, the level of significance was set at 5% and only responses with at least 10 responses deferred from the median response were included as dependent variables.

## Results

### Response rate and demographics

A total of 195 completed questionnaires were returned, giving a response rate of almost 20%. Thirty-six respondents stated that they did not lose their tap water during the incident. These were excluded from analysis, yielding a total sample size of 159 affected consumers.

To check for sampling bias, we compared the demographic characteristics of our respondents with those for Gloucestershire residents using the 2001 census survey (Table 1)[21]. Overall, our sample is representative. There is, however, an under-representation of younger and male participants, which is a common pattern in postal questionnaire studies [22]. There is also a disproportionate number of home owners and professionals versus people with elementary skills (e.g. farmers, porters, cleaners), which ties in with response rates typically being higher in the higher social classes than in the lower classes [23].

**Table 1:** Demographic comparison of study respondents with the 2001 census for Gloucestershire.

	n	Study population % (LCI - UCI)	Census population %
**Gender**			
male	62	40.0 (32.2 - 48.2)	48.8*
female	93	60.0 (51.8 - 67.8)	51.2*
**Age**			
29/30 or younger^**a**^	10	6.3 (3.1 - 11.3)	16.7*
30/31 to 59/60^**b**^	83	52.5 (44.4 - 60.5)	42.4*
60/61 or older^**c**^	65	41.1 (33.4 - 49.2)	22.4*
**Ethnicity**			
white UK	150	96.8 (92.6 - 98.9)	95.6
white non-UK	3	1.9 (0.4 - 5.6)	1.6
non-white	2	1.3 (0.2 - 4.6)	2.8
**Home ownership**			
sole/joint owner	138	87.3 (81.1 - 92.1)	74.3*
renting from council	13	8.2 (4.5 - 13.7)	8.5
renting privately	7	4.4 (1.8 - 8.9)	8.3
**Occupation**^**d**^			
managers & senior officials	14	15.1 (8.5 - 24.0)	15.3
professional	24	25.8 (17.3 - 35.9)	10.9*
associate prof. & technical	12	12.9 (6.8 - 21.5)	13.8
admin & secretarial	21	22.6 (14.6 - 32.4)	13.6*
skilled trade	8	8.6 (3.8 - 16.2)	12.4
personal service	4	4.3 (1.2 - 10.6)	6.5
sales & customer service	4	4.3 (1.2 - 10.6)	7.1
process & machine operatives	4	4.3 (1.2 - 10.6)	8.9
elementary	2	2.2 (0.3 - 7.6)	11.5*

^a ^Comprises our categories 20 or younger and 21-30 with the census categories 0-4, 5-9, 10-14, 15-19, 20-24 and 25-29.

^b ^Comprises our categories 31-40, 41-50 and 51-60 with the census categories 30-34, 35-39, 40-44,45-49, 50-54 and 55-59.

^c ^Comprises our categories 61-70 and 71 or older with the census categories 60-64, 65-69, 70-74,75-79, 80-84 and 85+.

^d ^Occupation data collected through an open-ended question was re-coded on a nine point Socio-Economic Scale adhering to the British Standard Occupational Classification 2000 [40] and using the Computer Assisted Structured Coding Tool [41].

*Census population estimate falls outside 95% confidence interval of census population.

### Participant experiences during the 2007 floods and participant backgrounds

During the 2007 floods, 33 respondents (20.9%) lost their main electricity and two (1.3%) were flooded. Twenty-seven participants (17.1%) chose to leave their homes due to the disaster. Of these, 23 stated that the main reason for not staying was difficulties managing without mains water, with several highlighting that they had (young) children or were 'vulnerable' due to ill health. Only one person left because of the floods, while no one stated loss of electricity or their home being flooded as their main reason.

Five households (3.2%) reported experience of flooding prior to the floods of 2007, but none of them were flooded during the Mythe incident. Forty-nine participants (31.6%) had previously experienced tap water loss; thirty-three of them recalled the cause being maintenance work and/or burst mains pipes, whereas only four had experienced water loss due to natural events such as floods or snow/ice. For the majority of these previous experiences (79.6%), participants could not recall if a water notice had been issued or they did not specify. Eight participants were certain that they did not receive a notice, whereas one participant recalled a 'Do Not Drink' notice and another a 'Do Not Use' notice.

In their general everyday life (i.e. outside the context of the incident), our participants show a very strong preference for tap water (124/151 (straight from the tap 92/151, boiled or filtered 32/151)), with only 16.5% preferring some type of bottled water and 1.3% stating that they drink other drinks than water.

### Information sources

Generally, information about the (imminent) loss of tap water reached consumers via the local radio station (i.e., BBC Radio Gloucestershire) or they were told by family/friends (30.7% and 30.0%, respectively (N = 140)). As Table 2 shows, those who did not find out about the planned shutdown of the tap water beforehand usually became aware when they turned on their taps and no water came out (13.6%). When the water came back on, three consecutive notices were issued by the authorities: 'Do Not Drink', 'Boil Water' and 'Water Safe'. Although the authorities had official water notice leaflets delivered to all households, only an average of 40% used them as an information source. Instead, information about the notices tended to reach consumers through other channels; notably, local radio remained the primary information source throughout these three stages: 61.1%, 55.7%, and 56.9%, respectively. Similarly, when participants were asked to rank the information sources in order of preference for the incident, local radio was ranked highest (53.4%, N = 118), followed by family/friends (11.9%), local newspapers (11.0%) and STW (by telephone or internet) (10.2%).

**Table 2:** Use of information sources for all four stages of the incident.

Information source	**'Water Loss'**^**a **^**(N = 140)**		'Do Not Drink' (N = 144)		'Boil Water' (N = 115)		'Water Safe' (N = 137)	
	n	%	n	%	n	%	n	%
I turned on the tap and no water came out^b^	19	13.6						
family/friend/neighbour	42	30.0	22	15.3	12	10.4	28	20.4
leaflet through the post^c^	8	5.7	66	45.8	44	38.3	51	37.2
local newspaper	4	2.9	53	36.8	32	27.8	40	29.2
national newspaper	0	0	6	4.2	0	0	2	1.5
local charity/volunteers	1	0.7	4	2.8	1	0.9	2	1.5
GP/nurse/health organisation	0	0	0	0	2	1.8	1	0.7
water company	0	0	60	41.7	41	35.7	48	35.0
TV	15	10.7	40	27.8	19	16.5	32	23.4
local radio	43	30.7	88	61.1	64	55.7	78	56.9
national radio	3	2.1	5	3.5	2	1.7	5	3.6
local government/council	0	0	3	2.1	3	2.6	1	0.7
website	1	0.7	15	10.4	8	6.9	7	5.1
other	4	2.9	3	2.1	4	2.5	3	2.2

^a ^For the 'Water Loss' stage, respondents could only choose one option (i.e. the information source first used), whereas for all other stages multiple responses were possible.

^b ^Option only available for the 'Water Loss' stage.

^c ^No leaflets were handed out during the 'Water Loss' stage.

While information source use did not differ much between the three notice stages, the total number of sources used did differ. The total number of information sources utilised during the 'Do Not Drink' stage was higher (M = 2.602, LCI = 2.320, UCI = 2.884) compared to the 'Boil Water' (M = 2.019, LCI = 1.792, UCI = 2.247) and 'Water Safe' (M = 2.146, LCI = 1.919, UCI = 2.372) stages, as confirmed by a repeated measures ANOVA with Bonferroni adjustment (F_(1) _= 10.747, p = .001).

In order to investigate whether our predictor factors were associated with consumers' choice of information source, we entered the information sources as well as the total number of information sources used during the incident into a MANOVA as dependent variables, with gender, age, home ownership and whether or not in paid employment as the independent variables. The final parameter estimates in the final analysis are shown in Table 3. The key observations are that increasing age and being in paid employment were negatively associated with using leaflets and using the local newspaper was marginally associated with increasing age. This would suggest that older consumers, in particular, but also those in paid employment do not seem to have used the leaflets. For example in the 'Do Not Drink' phase 43 of 83 (52%) respondents under 60 years reported using the leaflet compared to only 23 of 60 (38%) of people over 60. In the 'Boil Water' phase, these responses were 30/71 (42%) and 14/44 (32%) respectively.

**Table 3:** Final parameter estimates of MANOVA of predictors of information source use

Dependent variable	Predictor variables	B	LCI	UCI	p
family/friend/neighbour	Intercept	0.139	-0.057	0.335	
	Age	0.024	-0.02	0.069	0.287
	paid employment	-0.094	-0.228	0.039	0.165
leaflet through the post	Intercept	0.884	0.638	1.129	
	Age	-0.113	-0.169	-0.057	0.0001
	paid employment	-0.239	-0.407	-0.071	0.006
local newspaper	Intercept	0.079	-0.166	0.324	
	Age	0.06	0.004	0.116	0.034
	paid employment	0.09	-0.078	0.257	0.292

### Advice recollection

Most consumers (75.6% (118/156)) were informed about the loss of tap water beforehand. The majority of consumers reported receiving tap water advice ('Do Not Drink': 138/155; 'Boil Water': 110/154; 'Water Safe':136/154), with 94 consumers (63.9%) reporting receiving all three notices. Several consumers reported not receiving advice about their tap water during one or more of the stages; notably, five consumers reported receiving no advice at all.

As Table 4 shows, there was a lot of confusion as to which water notices were in place during the 'Do Not Drink' and 'Boil Water' stages, with correct recall varying from 23.2% (33/142) to 26.7% (31/116). During the 'Do Not Drink' stage, most consumers believed two notices were in place at the same time; alternatively the 'Do Not Drink' notice was confused with 'Do Not Use' and 'Boil Water'. Similarly, half of consumers - males in particular - recalled that when the first notice was changed, the new advice was that the water was safe.

**Table 4:** Advice recollection for the 'Do Not Drink' and 'Boil Water' stages.

	'Do Not Drink' (N = 142)		'Boil Water' (N = 116)	
	n	%	n	%
there was one advice: do not use	23	16.2	1	0.9
there was one advice: do not drink	33	23.2	9	7.8
there was one advice: boil	24	16.9	31	26.7
there was one advice: safe	2	1.4	58	50.0
not sure what the advice was	10	7.0	9	7.8
there was more than one type of advice	50	35.2	8	6.9

We queried how clear the advice about tap water was using a five-point Likert scale (1 = 'very unclear', 2 ='unclear', 3 ='understandable', 4 ='clear', and 5 ='very clear'). Twenty-three consumers (16.3%) stated that the advice was 'very clear', 59 (41.8%) that it was 'clear' and a further 41 (29.1%) felt that it was 'understandable' (N = 141). Similarly, a four-point Likert scale (1 ='very uninformed' - 4 ='very informed') revealed that the majority of consumers felt 'informed' (97/142) or 'very informed' (22/142). Not surprisingly, consumers who left their homes during the crisis consistently gave lower ratings for clarity of advice and feeling informed.

We used a MANOVA to test the association between information source use (i.e. family/friends/neighbour, leaflet through the post, local newspaper, water company, TV, local radio, and the number of information sources used) and clarity of advice and feeling informed. The final model is shown in Table 5. Use of local newspapers as an information source was positively associated with increased clarity of advice. Water company use was associated with feeling informed. The association between use of the local radio and feeling informed was approaching significance.

**Table 5:** Final parameter estimates of MANOVA of predictors of clarity of advice and feeling informed

Dependent variable	Predictor variables	B	LCI	UCI	p
clarity of advice	Intercept	3.204	2.894	3.513	
	local newspaper	0.559	0.134	0.983	0.010
	water company	0.278	-0.111	0.668	0.160
	local radio	0.232	-0.142	0.606	0.222
feeling informed	Intercept	2.627	2.406	2.848	
	local newspaper	0.193	-0.11	0.496	0.210
	water company	0.314	0.036	0.592	0.027
	local radio	0.255	-0.012	0.522	0.061

### Water access and preferences

For the incident as a whole, 21 of the 69 participants who used temporary water supplies reported serious problems securing access to temporary water supplies. Access failures were mainly due to empty bowsers and/or no available stock of bottled water (n = 15). Other reasons included problems travelling to and from the water sites (n = 6) or not being able to locate them (n = 7). Except for one participant who had to rely solely on water supplied by family/friends, consumers did on at least one occasion gain access to temporary water supplied by STW.

During the 'Water Loss' stage, collecting bottled water from distribution sites was the most favoured means of procuring water (84.9%, N = 159), compared to bowser water (57.9%). In addition, 35.8% of consumers bought bottled water, or collected water from family/friends (8.2%). Consumers who found out about the water loss beforehand tended to refrain from buying bottled water, instead opting for supplied bottled water. Consumers who generally (i.e. outside the context of the incident) prefer to drink water straight from the tap also tended to refrain from buying bottled water, with many of them choosing to get water from family/friends in unaffected areas.

For the 'Do Not Drink' and 'Boil Water' stages, consumers were asked to rank different types of drinking water in terms of frequency of use. Based on these data, we can discern popularity in terms of how many respondents used a particular source as well as its mode rank and the total number of rank 1s that it received (Table 6). Both in terms of users and ranks, tap water was the least preferred water source for both stages, including tap water from family/friends, while bottled water was clearly the favoured type of drinking water. Although many households did collect water from bowsers for drinking purposes, rank data shows it to be quite unpopular.

**Table 6:** Water drinking preference for the 'Do Not Drink' and 'Boil Water' stages.

	'Do Not Drink' (N = 155)			'Boil Water' (N = 108)		
Popularity of water sources	No of Users	Mode rank	Rank 1s (%)	No of Users	Mode rank	Rank 1s (%)
bottled water from distribution site	133	1	70.0	90	1	69.9
bought bottled water, still	73	1	20.0	46	2	16.3
water from bowsers	34	2	3.8	18	2	3.3
bought bottled water, sparkling	21	2	2.3	9	2	1.1
chilled boiled tap water	17	2	0.0	8	2	2.2
water straight from the tap	13	8	0.8	21	2	3.3
tap water from family/friends	12	1	3.1	4	1	2.2
filtered tap water	8	7	0.0	9	1	2.2

We also found that 28 participants (17.6%) carried on using temporary water sources after the tap water was safe, because they were not convinced that it was safe, and some also commented that the water was dirty, cloudy and smelly, or tasted strange.

### Tap water behaviour and compliance

Participants were asked to specify their use of tap water during the 'Do Not Drink' and 'Boil Water' stages (Table 7). Of particular concern is the high proportion of consumers who ingested (i.e. brushed teeth, prepared food/cook and drunk) unboiled tap water; a risky behaviour which increased substantially when the advice changed to 'Boil Water'. Ingestion of boiled water during the 'Do Not Drink' stage was also risky and prominent. Table 7 also reveals that some consumers refrained from using tap water for safe actions such as hand washing, showering/bathing and toilet flushing, or they used boiled tap water for these purposes. Thus, individuals' tap water actions commonly included both risky and over-cautious behaviour.

**Table 7:** Use of tap water during the 'Do Not Drink' and 'Boil Water' stages.

	'Do Not Drink' (N = 159)		'Boil Water' (N = 116)		p
	n	%	n	%	
**Using unboiled tap water for**					
flush toilet	148	93.1	105	90.5	0.439
shower/bathe	120	75.5	97	83.6	0.102
wash hands	83	52.2	81	69.8	0.003
prepare/cook food with^a^	34	21.4	49	42.2	2.0 × 10^-4^
brush teeth^a^	26	16.4	44	37.8	4.9 × 10^-5^
Drink^a^	15	9.4	34	29.3	2.1 × 10^-5^
**Using boiled tap water for**					
flush toilet	4	2.5	5	4.3	0.409
shower/bathe	6	3.8	5	4.3	0.823
wash hands	17	10.7	6	5.2	0.103
prepare/cook food with^b^	75	47.2	41	35.3	0.050
brush teeth^b^	61	38.4	29	25.0	0.020
drink hot^b^	67	42.1	45	38.8	0.577
drink cold^b^	34	21.4	23	19.8	0.753

^a^Action not safe with unboiled water if a 'Do Not Drink' or Boil Water' notice is in place.

^b^Action not safe with boiled water if a 'Do Not Drink' notice is in place.

Regarding compliance with water advice about drinking, 47.2% (75/159; based on respondents drinking water straight from the tap and/or boiled tap water during the 'Do Not Drink' stage) versus 29.3% (34/116; based on respondents drinking unboiled tap water during the 'Boil Water' stage) of respondents did not comply with the notices. Looking at overall compliance with water advice, i.e., including brushing teeth and preparing/cooking food, non-compliance increases to 62.9% (100/159) and 48.3% (56/116) for the 'Do Not Drink' and the 'Boil Water' notices, respectively. The higher non-compliance rates for 'Do Not Drink' are pre-dominantly attributed to the high use of boiled tap water.

In order to test our hypothesis that non-compliance would be greater for 'Do Not Drink', we used a generalised estimating equation with drinking water compliance and overall compliance as dependent variables and with incident stage, demographics and participants' general drinking water preference and previous experience of loss of tap water as predictor variables. The final model is shown in Table 8. It can be seen that the main factor associated with both drinking water compliance and overall compliance is incident stage (i.e. 'Do Not Drink' stage versus 'Boil Water' stage), confirming our hypothesis. Drinking water compliance was, in addition, associated with employment, i.e. those in paid employment were less likely to comply. There was some suggestion that females were more likely to be compliant, but this association was not significant.

**Table 8:** Final parameter estimates of generalised estimating equation of predictors of compliance with water advice

Dependent variables	Predictor variables		B	LCI	UCI	p
drinking water compliance	incident stage	Do Not Drink	1			
		Boil Water	1.35	1.11	1.64	0.002
	paid employment	No	1			
		Yes	0.81	0.68	0.97	0.024
	gender	Male	1			
		Female	1.154	0.96	1.40	0.137
overall compliance	incident stage	Do Not Drink	1			
		Boil Water	1.79	1.34	2.34	2.3 × 10^-5^

Furthermore, respondents' reported use of unboiled and boiled tap water during the 'Do Not Drink' and 'Boil Water' stages was analysed by rotated Equamax factor analysis followed by generalised estimating equation analysis with demographics, sources of information used and participants' general drinking water preference and previous experience of loss of tap water as predictor variables. No significant associations were identified.

### Bowser water behaviour and compliance

Ingestion of unboiled bowser water was limited (Table 9). In contrast to tap water compliance, only six of the 53 participants (11.3%) who utilised bowser water for drinking during one or more stages of the incident failed to comply with the advice to boil it before drinking it, while overall non-compliance was 27.3% (21/77). Numbers were insufficient to determine factors associated with compliance with advice on bowser water.

**Table 9:** Use of unboiled bowser water during the first three stages.

Use of unboiled bowser water	**n/N**^**a**^	%
prepare/cook food with^b^	16/72	22.2
brush teeth^b^	11/47	23.4
drink cold^b^	5/50	10.0
drink hot^b^	5/31	16.1

^a ^The number of participants who said they used unboiled bowser water (n) out of the number of participants who used boiled and/or unboiled bowser water (N). In total, 77 participants used bowser water for some purpose, but not all of them replied to each bowser water question.

^b ^Action not safe.

## Discussion

The world faces unprecedented changes in climate and environment, and an increased risk of natural disasters, which for the UK will include floods, droughts and heat waves [24]. Natural disasters, whether hurricanes or floods, potentially pose a risk to drinking water and consequently public health. After Hurricane Ami hit an island of Fiji, nearly 75% of water samples taken showed contamination [25]. Due to scarcity of safe water during large emergencies, high incidences of fever, diarrhoeal illnesses and skin infections are common place, at least in low income countries [26]. Isolation from medical care further increases rates of morbidity and mortality. Where drinking water is still available, albeit not safe to drink (with or without treatment), water advice is issued. Public health communication is key to safe water behaviour and compliance with advice.

Compliance studies have varied in methodology with some focusing on unboiled tap water and others on boiled water, reducing comparability across studies and across notices. With the exception of the Hurricane Rita study [13], previous studies have focused on human error and everyday incidents, and for the absolute majority of these, the notice investigated is 'Boil Water'. This study is the first to contrast advice recollection, information use, water behaviour and compliance during an incident involving two notices. It is also the first to investigate a 'Do Not Drink' notice issued due to a natural disaster, and the first to include compliance with the general advice to boil bowser water.

It should be noted that the study took place 18 months after the incident. This may have resulted in a lower than normal response rate. Compliance surveys sent out within one month of the incident have yielded response rates around 65% [e.g. [12]]. However, risk perception studies normally attract response rates just below 20% [e.g. [27]]. The time delay may also have impacted consumers' recall of actions and events. However, the 2007 Gloucestershire flood incident was unique and highly memorable; our study has shown that consumers' main concern revolved around their tap water; and in focus groups carried out after the postal questionnaire, consumers shared very detailed accounts of the incident and their quest to access safe drinking water. The time lag, nevertheless, does place some restrictions on the results and conclusions presented here.

Consumers relied on a range of sources for information about safe water behaviour, and use of sources differed somewhat between the first and the subsequent three stages, with the exception of local radio which remained the primary source through the whole incident. Information usage was at its highest during the 'Do Not Drink' notice stage. In situations of lots of uncertainty, the public commonly seeks out more information sources [27].

During natural disasters, water advice has commonly not reached consumers. In the aftermath of Hurricane Rita, as few as 31% of people issued with a 'Boil Water' notice were aware of it [13]. In the present study, 3% of participants reported not receiving any water advice at all when the tap water was returned. In addition, several consumers reported not receiving one of the advices. Due to the nature of the crisis, Severn Trent Water was not able to inform consumers before the water supply failed. Similarly, printed media and media websites were unable to deliver advance warnings. Official water notices were, however, issued during the three subsequent stages. These were consulted by only about 40% of consumers. Notably, older participants and those in paid employment hardly used them. For an information source that did reach all households, a 40% efficacy is very worrying, and it is essential that we evaluate how official advice is provided to the general public; especially since we also found that receiving information from the water company was positively associated with feeling informed. The apparent discrepancy most likely stems, in part, from the fact that the first water notice was not a standard water industry notice; thus, it is therefore essential that standard water industry protocol be upheld in future incidents. This is also the recommendation of the Drinking Water Inspectorate in their Incident Assessment Letter [20]. Another reason may be the close collaboration between Severn Trent Water and local media, which we discuss further below.

As printed information could not be made available when the tap water was turned off, family/friends and direct media such as radio and television featured prominently during the first incident stage. In New South Wales, 67% of people found out about severe storm warnings through television, but during the storm they relied more on radio and family/friends [28].

Personal dissemination networks have been shown to be particularly vital for vulnerable sub-populations [29], and interpersonal information is often perceived as more credible and efficient than official information sources [10,30]. Decisions on how to respond to flood warnings have also been found to rely to some extent on the behaviours and attitudes of people close by [31]. In the present study, we found that family/friends/neighbours were the second most preferred source along with local newspapers (which was significantly used by older consumers) and the water company, despite a drop in use after the initial stage which presumably is due to the availability of printed media from then onwards. Dissemination plans should be revised in order to tap into family/friends/neighbours as a potential information stream, e.g. personal networks and nomination of local 'disaster contact persons' can be established through local community organisations, and dissemination through these networks should be given prominence.

Reflecting the complexity of the event and the combination of two subsequent notices, consumers' advice recollection was noticeably affected, leaving consumers highly uncertain about which notice was in place when, with significant proportions believing two notices were in place at the same time. Clearly, the public is not aware of the exclusive nature of notices (i.e. only one can be in place at a time) or that there are several different types of notices. This could indicate that they construe water as either safe or not safe.

Typically, non-compliance with 'Boil Water' advice ranges between 9% and 20% [11,32] whereas after Hurricane Rita, only one-third reported boiling water for drinking [13]. If unsafe actions such as using unboiled water for brushing teeth or preparing/cooking food are considered, non-compliance increases dramatically to 57% and 77% for human error and natural disasters, respectively [13,32]. Our data show high non-compliance for the 'Boil Water' notice (29.3%), and when including other unsafe behaviours, it rises to 48.3%. Comparison of 'Boil Water' compliance for this incident with preliminary results from a recent UK human error 'Boil Water' incident tentatively confirms that natural disasters are associated with higher degrees of non-compliance compared to human error incidents (Knapton, Hunter & Rundblad; submitted 2010).

The first water notice issued was 'Do Not Drink'. To date there is only one previous study of this advice, a human-error incident in Israel in 2001. Non-compliance was estimated to 18% [33]. We found that non-compliance for drinking was significantly higher for the 'Do Not Drink' notice (47.2%) compared to the 'Boil Water' notice (29.3%). Similarly, overall non-compliance was also significantly higher: 62.9% versus 48.3%. By contrasting participants' use of unboiled and boiled tap water, we can see for the first time that the higher use of contaminated water during the 'Do Not Drink' notice stage is due to consumers boiling the water, presumably due to a belief that it renders it safe to drink and use. These results are in line with Rundblad's [34] hypothetical 'Do Not Drink' scenario study which predicted that 39.3% of consumers would boil and drink contaminated water. In the Mythe incident, there were attempts to neutralise this common folk belief by including additional information on notices and in material supplied to media and the public. It is beyond the scope of this study to evaluate if this addition did impact consumer behaviour; however as the high degree of non-compliance here suggests, this measure alone is far from sufficient. It is highly likely that this folk belief in boiling is linked to the aforementioned binary folk classification of water. The higher degree of non-compliance for 'Do Not Drink' is not because consumers acted differently for the two notices, but rather that the protective measures they took were more or less the same, and as such, they were not sufficient for the more restrictive notice.

It should be noted that many consumers varied in their behaviour such that they displayed a mixture of safe and unsafe behaviours. Some also engaged in over-cautious behaviour such as flushing the toilet with boiled tap water or avoiding to flush. The mixture of risky and over-cautious actions can be attributed to consumers not being aware or being unsure of what actions are unsafe. The title of the notice 'Do Not Drink' is highly misleading as it fails to highlight all ingestion actions listed on the notice [34]; an alternative title, such as 'External Use Only' could prove more informative. In addition, the Drinking Water Inspectorate were strongly critical of the notice issued by the health authorities because it did not follow water industry standards and the messages were unclear [20]. We also found that 17.6% carried on using temporary water supplies after the tap water had been declared safe, which is similar to the 10% of consumers continuing to boil water after the 'Boil Water' notice was lifted in Willocks and colleagues' study of 2000 hospital employees in the North Thames region, UK, in 1997 [32]. Information and reassurance that water is safe or that certain water actions are safe during a particular notice are as important from a health point of view as informing about unsafe actions. Worries about potential health hazards can cause severe anxiety and, like detection of discolouration and odour in the water, causes increases in reports of symptoms of waterborne illness [35]. It is vital that the titles of notices be reviewed and that the public's classification of water and beliefs about precautionary actions such as boiling be addressed through public health education.

It is important to separate the public's perception of information sources and their behaviour. While media has been found to impact general risk perceptions because these perceptions are impersonal, they do not necessarily impact the personal risk perceptions that would initiate behaviour responses [36]. In the present study, media did not affect consumer behaviour. However, BBC Radio Gloucestershire came to take up a rather unique position. Throughout the incident, it remained the primary information source, both in terms of usage and preference, and its association with feeling informed was approaching significance. BBC Radio Gloucestershire estimates that roughly half the population of Gloucestershire kept up-to-date about the crisis by listening in, and their website saw a 159% increase of users (personal communication). We believe that its apparent success rested on more than radio being easily accessible. Other local mass media was also favoured; notably, local newspapers were positively associated with clarity of advice. This could indicate a need for geographic-specific information [28]. Severn Trent Water followed standard water industry practice and issued frequent and regular bulletins to the local media in parallel to issuing notices. BBC Radio Gloucestershire was quick to broadcast not only about the mains water loss, but also about locations of distribution sites. Users of local radio therefore knew there was no need to buy bottled water. Another important step taken was the broadcasting of the daily press conferences with updates from Severn Trent Water and Gloucestershire Constabulary. Altogether, BBC Radio Gloucestershire was able to establish themselves as a timely and trustworthy information source. Building on the example of BBC Radio Gloucestershire, it is essential that local media be pre-prepared and, during an event, be continuously up-dated to maximise their role in ensuring public safety.

Although a potential key player in public behaviour response, no information source was associated with water behaviour. We did, however, find that those in paid employment were significantly less likely to comply with drinking water advice. Demographic factors such as socioeconomic status have previously been found to influence behaviour during natural disasters [37-39]. In order to ensure appropriate and safe behaviour during natural disasters, public health education needs to reach all income quartiles, at home or at work.

Lack of electricity contributed to non-compliance after Hurricane Rita [13]. In this incident, approximately 20% of our participants were without electricity, but loss of mains electricity occurred at the same time as consumers were without main tap water and only lasted for 24 hours. Consequently, electricity could only have impacted boiling of bowser water. However, compliance with bowser water advice was very high: 88.7% (drinking) and 72.7% (including food preparation, etc.) Bowser advice is more consistent and thus less open to interpretation as it is water industry practice for every bowser to bear a clear permanent 'Boil Water' message at the point where consumers draw water. Even so, whether consumers conceptualised the instruction to boil the water as relating to use of unsterilised collection vessels rather than unsafe bowser water could not be ascertained in the present study.

Whilst bowser water was used, it was not popular for drinking. Instead, bottled water was preferred during both notices. It has been suggested that bowser water and bottled water serve slightly different purposes, with the former to be used for personal hygiene and cooking, and the latter for drinking. However, it was not clear from the study whether consumers understood such a distinction, or whether there is a general distrust in the quality of bowser water. Thus in future incidents, bottled water should be considered a priority over bowser water, where supply, distribution and recycling allows. More than half of consumers bought bottled water even though the Security and Emergency Measures Direction 1998 requires all water companies to provide a minimum of 10 litres of water per person per day in case of supply failure [15]. We trace buying of bottled water to the public not being aware of this provision duty, especially since consumers who found out about the water loss beforehand tended to refrain from buying bottled water. It is also possible that consumers did not trust the water company to supply enough water and in time.

## Conclusion

A high proportion of consumers, especially elderly, reported not having used the official leaflets containing advice on safe and unsafe water behaviour. Instead, local media and family/friends functioned as main information channels. Contacts with local media and community/personal networks should be established, maintained and kept up-to-date with drinking water standards and emergency protocol, so that when an incident occurs official advice can reach all consumers of all demographic backgrounds in a timely fashion. High degrees of non-compliance, especially for the 'Do Not Drink' notice, are the result of consumers employing insufficient protective measures, presumably due to incorrect folk beliefs regarding water contaminants and boiling. Unsafe behaviour was commonly coupled with over-cautious behaviour illustrating that consumers are equally unaware of what actions are safe. Current public health education provision should be evaluated and drinking water knowledge be included, in order to minimise risky behaviour and avoid unnecessary stress from over-caution during incidents.

## Competing interests

PRH is Chair of Science Board to Suez Environment, Paris, Chair of Board of Directors of Institute of Public Health and Water Research, and acted as consultant to Danone Beverages. All other authors have no competing interests to declare.

## Authors' contributions

GR designed the study, contributed to literature review, contributed to questionnaire design, participated in data analysis, wrote the manuscript. OK contributed to literature review, contributed to questionnaire design, participated in data analysis, contributed to manuscript. PRH contributed to study design, participated in data analysis, contributed to manuscript. All authors read and approved the final manuscript.

## Pre-publication history

The pre-publication history for this paper can be accessed here:

http://www.biomedcentral.com/1471-2458/10/641/prepub

## References

[B1] Glik DC Risk communication for public health emergencies Annu Rev Public Health 2007 28 33 54 10.1146/annurev.publhealth.28.021406.144123 17222081

[B2] Brodie M Weltzien E Altman D Blendon RJ Benson JM Experiences of Hurricane Katrina evacuees in Houston shelters Am J Public Health 2006 96 8 1402 1408 10.2105/AJPH.2005.084475 16571686 PMC1522113

[B3] Rogers MB Amlot R Rubin GJ Wessely S Krieger K Mediating the social and psychological impacts of terrorists attacks: the role of perception and risk communication International Review of Psychiatry 2007 19 3 279 288 10.1080/09540260701349373 17566905

[B4] Hunter PR Reid M Thompson KC, Gray J Poor communication during a contamination event may cause more harm to public health then the actual event itself Water Contamination Emergencies 2006 Cambridge: RSC Publishing 156 164

[B5] Renn O Kasperson RE, Stallen PJ Risk Communication and the social amplification of risk Communicating Risks to the Public: International Perspectives 1991 Amsterdam and New York: Kluwer Academic 287 324

[B6] Alaszewski A Risk communication: identifying the importance of social context Health, Risk & Society 2005 7 2 101 105

[B7] Griffin RJ Dunwoody S Zabala F Public reliance on risk communication channels in the wake of a cryptosporidium outbreak Risk Anal 1998 18 4 367 375 10.1111/j.1539-6924.1998.tb00350.x 9775446

[B8] Doria MF Abubakar I Syed Q Hughes S Hunter PR Perceived causes of sporadic cryptosporidiosis and their relation to sources of information BMJ 2006 60 745 750 10.1136/jech.2005.041731 PMC2566020 16905716

[B9] Hunter PR Syed Q Community surveys of self-reported diarrhoea can dramatically overestimate the size of outbreaks of waterborne cryptosporidiosis Water Science and Technology 2001 43 12 27 30 11464765

[B10] Griffin RJ Dunwoody S The relation of communication to risk judgment and preventive behavior related to lead in tap water Health Communication 2000 12 1 81 107 10.1207/S15327027HC1201_05 10938908

[B11] O'Donnell M Platt C Aston R Effect of a boil water notice on behaviour in the management of a water contamination incident Communicable Disease and Public Health 2000 3 1 56 59 10743321

[B12] Karagiannis I Schimmer B de Roda Husman AM Compliance with boil water advice following a water contamination incident in the Netherlands in 2007 Eurosurveillance 2009 14 12 8 10 19341604

[B13] Ram PK Blanton E Kinghoffer D Platek M Piper J Straif-Bourgeois S Bonner MR Mintz ED Household water disinfection in hurricane-affected communities of Louisiana: Implications for disaster preparedness for the general public Am J Public Health 2007 97 Suppl 1 S130 S135 10.2105/AJPH.2006.094441 17413065 PMC1854986

[B14] Pitt M Lessons from the 2007 floods: what people need 2008 London: Cabinet Office

[B15] Severn Trent Water The Impact of the July Floods on the Water Infrastructure and Customer Service 2007 Gloucestershire

[B16] Knight K Facing the challenge. London 2008

[B17] Gloucestershire Constabulary Operation Outlook narrative and analysis: Chief Constable's report to the Gloucestershire Police Authority 2008 Gloucestershire

[B18] Drinking Water Inspectorate Incidents in England and Wales 2006. London 2007

[B19] Drinking Water Inspectorate Incidents in England and Wales 2007. London 2008

[B20] Drinking Water Inspectorate, Health Protection Agency Drinking water safety - Guidance to health and water professionals. London 2009

[B21] Census 2001 http://www.gloucestershire.gov.uk/inform/index.cfm?articleid=94726

[B22] Murray SA Graham LJC Practice based health needs assessment: use of four methods in a small neighbourhood BMJ 1995 310 1443 1448 7613279 10.1136/bmj.310.6992.1443 PMC2549816

[B23] Sheikh K Mattingly S Investigating non-response bias in mail surveys J Epidemiol Community Health 1981 35 4 293 296 10.1136/jech.35.4.293 6461711 PMC1052180

[B24] van Aalst MK The impacts of climate change on the risk of natural disasters Disasters 2006 30 1 5 18 10.1111/j.1467-9523.2006.00303.x 16512858

[B25] Mosley LM Singh S Sharp DS Effects of a tropical cyclone on the drinking-water quality of a remote Pacific island Disasters 2004 28 4 405 417 10.1111/j.0361-3666.2004.00266.x 15569381

[B26] Rashid SF The urban poor in Dhaka City: their struggles and coping strategies during the floods of 1998 Disasters 2000 24 3 240 253 10.1111/1467-7717.00145 11026157

[B27] ter Huurne E Gutteling J Information needs and risk perception as predictors of risk information seeking Journal of Risk Research 2008 11 7 847 862 10.1080/13669870701875750

[B28] Cretikos M Eastwood K Dalton C Merritt T Tuyl F Winn L Durrheim D Household disaster preparedness and information sources: rapid cluster survey after a storm in New South Wales, Australia BMC Public Health 2008 8 195 203 10.1186/1471-2458-8-195 18533010 PMC2443139

[B29] Spence PR Lachlan K Burke J Seeger MW Media use and information needs of the disabled during a natural disaster J Health Care Poor Undeserved 2007 18 394 404 10.1353/hpu.2007.0047 17483567

[B30] Parker DJ Handmer JW The role of unofficial flood warning systems Journal of Contingencies and Crisis Management 1998 6 1 45 60 10.1111/1468-5973.00067

[B31] Drabek TE Parker DJ The social factors that constrain human responses to flood warnings Floods 2000 1 London: Routledge 361 376

[B32] Willocks LJ Sufi F Wall R Seng C Swan AV Compliance with advice to boil drinking water during an outbreak of cryptosporidiosis Communicable Disease and Public Health 2000 3 2 137 138 10902259

[B33] Winston G Lerman S Goldberger S Collins M Leventhal A A tap water turbidity crisis in Tel Aviv, Israel, due to technical failure: toxicological and risk management issues International Journal of Hygiene and Environmental Health 2003 206 193 2000 10.1078/1438-4639-00206 12872527

[B34] Rundblad G The semantics and pragmatics of water notices and the impact on public health Journal of Water and Health 2008 6 Suppl 1 77 86 10.2166/wh.2008.130 18401131

[B35] Fowle SE Constantine CE Fone D McCloskey B An epidemiological study after a water contamination incident near Worcester, England in April 1994 J Epidemiol Community Health 1996 50 1 18 23 10.1136/jech.50.1.18 8762348 PMC1060198

[B36] Wåhlberg AA Sjöberg L Risk perception and the media Journal of Risk Research 2000 3 1 31 50 10.1080/136698700376699

[B37] Blendon RJ Benson JM DesRoches CM Lyon-Daniel K Mitchell EW Pollard WE The public's preparedness for hurricanes in four affected regions Public Health Rep 2007 122 167 176 17357359 10.1177/003335490712200206 PMC1820441

[B38] Tierney K Lindell MK Perry R Facing the Unexpected 2001 London: Altamira Press

[B39] Phillips BD Metz WC Nieves LA Disaster threat: preparedness and potential response of the lowest income quartile Environmental Hazards 2005 6 123 133 10.1016/j.hazards.2006.05.001

[B40] Office for National Statistics: Standard Occupational Classification 2000 http://www.ons.gov.uk/about-statistics/classifications/archived/SOC2000/index.html

[B41] Warwick Institute for Employment Research: CASCOT http://www2.warwick.ac.uk/fac/soc/ier/publications/software/cascot/

